# The predictive value of nasolacrimal sac biopsy in endoscopic dacryocystorhinostomy

**DOI:** 10.1016/j.amsu.2021.102317

**Published:** 2021-04-16

**Authors:** Ibrahim Eldsoky, Wael Fawzy Ismaiel, Abdulkarim Hasan, Mohamed Hussein Abdelazim, Ahmed Abd Alrahman Ibrahim, Mahmoud Elsaid Alsobky, Ahmed Rabie Mohammed

**Affiliations:** a ENT Department, Faculty of Medicine, Al-Azhar University, Cairo, Egypt; b ENT Department, Faculty of Medicine, Al-Azhar University, Damietta Branch, Egypt; c Pathology Department, Faculty of Medicine, Al-Azhar University, Cairo, 11884, Egypt; d Ophthalmology Department, Faculty of Medicine, Al-Azhar University, Cairo, Egypt

**Keywords:** Chronic dacryocystits, Endoscopic DCR, Epiphora, Histopathology, Nasolacrimal sac

## Abstract

**Background:**

During dacryocystorhinostomy (DCR), the lacrimal sac wall biopsy is not routinely performed in our hospital, but it is recommended if there is a suspicion of underlying disease other than preoperatively or intraoperatively chronic inflammation.

**Objective:**

Most of patients with epiphora have different causes of nasolacrimal duct obstruction (NLDO). This study aims to examine how important routine lacrimal sac biopsy is during endoscopic DCR surgery.

**Patients & methods:**

The study included 50 patients with chronic unilateral epiphora. All patients underwent endoscopic DCR with NLD biopsy. Histopathologic analysis was performed for each specimen.

**Results:**

The findings of NLD biopsy showed chronic inflammation in 33 cases (66%), chronic dacryocystitis in 9 cases (18%), dacryolith with dacryocystitis in one case, granuloma in 4 cases (8%), rhinoscleroma in 2 cases (4%), and one case had a neoplasm. Histopathologic findings were inflammatory cellular infiltrates in 56%, 30% and 14% in mild, moderate and severe chronic inflammatory state (CIS) score, respectively. Fibrosis in 18%, 20% and 62% in mild, moderate and severe CIS score, respectively. Capillary proliferation in 64%, 32% and 4% in mild, moderate and severe CIS score, respectively. Chronic inflammatory signs in 64%, 32% and 4% in mild, moderate and severe CIS score, respectively.

**Conclusion:**

Although neoplasm and granuloma are rare cause of lacrimal sac or duct obstruction requiring DCR, they were detected through nasolacrimal assessment and routine intraoperative lacrimal sac biopsy.

## Highlights

• Nasolacrimal sac biopsy in all endoscopic dacryocystorhinostomies is highly advised.
• Seven patients out of 50 revealed unexpected pathology results.
• Neoplasm and granuloma are detected in lacrimal sac or duct obstruction.

## 1 Introduction

Lacrimal canal and sac disorders that cause epiphora, punctual discharge, or medial canthal swelling, are common eye problems which make up around 3% of clinic visitors in some series [1]. In a pathologically closed lacrimal drainage system, obstruction of the nasolacrimal duct, whichever the cause, leads to an accumulation of tears with mucoid secretions and desquamated cells above the obstruction [2]. This creates a favorite environment for different bacterial species and result in dacryocystitis with its comorbidities [3]. Dacryocystitis is detected in cases of continuous lacrimation and detection of mucoid or mucopurulent discharge by pressure on the lacrimal sac, or detection of mucoid or mucopurulent discharge [4].

The DCR operation, a reliable, efficient, and well-established standard surgical operation for the management of partial or full nasolacrimal obstruction for reduction of epiphora and offering symptom relief to patients, is used to treat primary acquired nasolacrimal drainage system obstruction. It is the technique of choice for suspected lacrimal system pathology whereas biopsy may be planned [2]. Even chronic inflammatory state with granuloma, may appear as masses and may indicate to systemic disease that appear in lacrimal sac biopsy [5].

A thickened and purulent or mucoid matter in the duct lumen is visible macroscopically in an inflamed lacrimal sac sample. Wall biopsy often revealed a non-specific chronic inflammation with fibrosis and wall thickening owing to lymphocytic infiltrates with follicular formation. The risk of over-pressing over the lacrimal sac ameliorates specific pathologies, especially malignancies which trigger a NLDO, even though very low, it always exists [6,7].

Despite the fact that this proposal is disputable, these discoveries have led to the suggestion that lacrimal sac biopsy samples should not be routinely sent for pathologic analysis during DCR surgery, but rather for unusual clinical introductions or intraoperative discoveries [2].

**Anderson et al.** [8] believed that during DCR, all lacrimal sac walls must be biopsied. Thus, it had been mentioned in the literatures regarding the importance of lacrimal sac biopsy during DCR as the literature presents this difference in views between “biopsy always” and “in suspicious sac” [9].

The objective of this research was to investigate the effect of routine lacrimal sac biopsy done at DCR surgery.

## 2 Patients and method

This is a prospective analysis was performed on 50 patients with chronic unilateral epiphora, all patients were recruited from Cairo and Damietta, Al-Azhar University Hospitals, Egypt from December 2019 to December 2020. They were selected from patients attending the ENT and Ophthalmology clinics in Cairo (Al-Hussein university hospital) and Damietta Al-Azhar University Hospital. Informed consent was taken from all patients after clarification of the specifics of the research and of the procedure to be done. The Bioethical research committee approval was obtained from Al-Azhar University Hospitals for this study and the study was registered on https://clinicaltrials.gov/ct2/show/NCT04793230.

Inclusion criteria: Male and female patients with chronic lacrimal duct obstruction attended the outpatient clinic of Al-Azhar university hospitals for treatment during the study period. Exclusion criteria: Recurrent cases with previous external DCR and Patients who did not complete the required medical follow up at our hospitals.

### 2.1 Study population

This study includes fifty patients with chronic epiphora due to chronic nasal duct obstruction seeking for treatment by endoscopic intranasal DCR. Their age ranged from (20–70) years from both genders. They were selected from patients attending the Ophthalmology and ENT clinics. Their referring ophthalmologist diagnosed all of the patients with lacrimal obstruction.

### 2.2 Methods

**Fig. 1:**
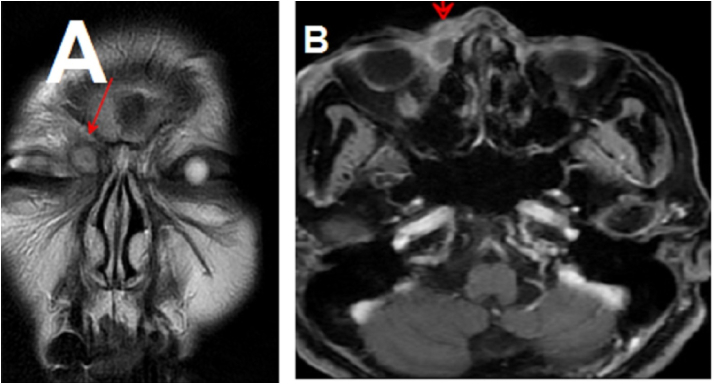
A&B: MRI orbit showing: Well defined cystic biloculated lesion seen implicating the medial inferior corner of the right orbit at the region of the lacrimal sac, it is seen extending into the right nasolacrimal duct.

Participants of both groups were subjected to the following preoperative measures:

1. Full history taking.
2. Ophthalmologic examination by ophthalmologist.
3. ENT examination including complete nasal examination.
4. Treatment of concomitant sinus disease, or reduction the probability of postoperative adherence.
5. The surgical site could be examined and cleaned more easily after surgery with a straight nasal septum.
6. Probing and syringing of the proximal lacrimal drainage up to the nasal wall, as well as lacrimal system irrigation, were used to assess lacrimal drainage anomalies for confirmation of NLDO.
7. Preoperative CT scan were performed in all cases and MRI orbit for some recurrent and selected cases. (Fig. 1).

### 2.3 Operative procedure

The procedure was carried out under general or local anesthesia. An otolaryngologist conducts the endoscopic intranasal operation, while an eye surgeon removes tissue from the sac and lacrimal tubes using lacrimal probes passed through the canaliculi. The whole operation is performed with a video camera connected to the nasal endoscope, which allows both surgeons to observe intranasal manipulations on a video monitor at the same time.

**Fig. 2:**
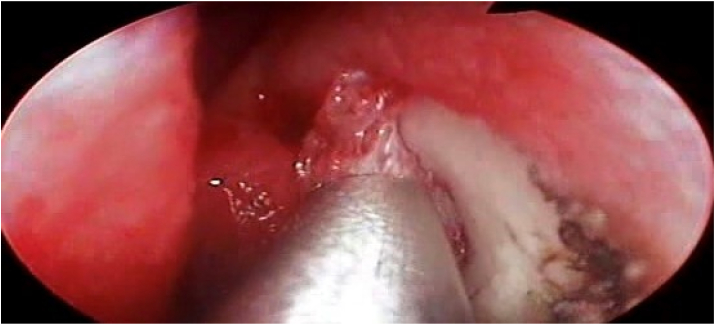
Incision through maxillary line with creation of mucosal flap and exposure of the lacrimal bone.

The lacrimal sac is situated just in front of the middle turbinate on the upper side of the lateral nasal wall. A lacrimal probe is inserted via a canaliculus and guided medially into the obstructed sac, where it tents the nasal mucosa. A sickle knife was used to start making a curvilinear incision in the nasal mucosa, roughly 1 cm anterior to the underlying tip of the probe, while a 0-or 30-degree nasal endoscope was used for visualization. Using a straight Blakesley forceps, the mucosal flap is lifted back and removed (Fig. 2). Additional scar tissue must often be removed in order to gain access to the sac's interior.

The lacrimal probe would have been revealed once the sac had been entered. An angled Blakesley forceps (Karl Storz Endoscopy, Culver City, California) aimed laterally is used to deepen the intranasal opening. The surgeon has to be careful to extract only the tissue that surrounds probe. With a 70-degree endoscope, direct visualization into the sac is frequently possible at this point. Once the intranasal opening is enlarged to almost 10 mm in diameter, the lacrimal probe must pass freely from the upper and lower canaliculi to the nose.

**Fig. 3:**
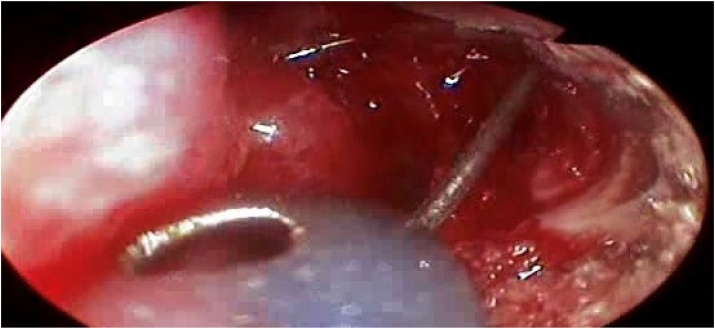
Stinting of the nasolacrimal duct after biopsy taking.

Subsequently, the lacrimal probe is substituted by a silicone rubber tube whose ends are stented over a rigid wire (Fig. 3), (Guibor Canaliculus Intubation Set, Concept Inc., Largo, Fla.). Forceps are used to grasp the tubing's ends and direct them out of the nose. They're tied and trimmed in such a way that the knot is inside the nose's cavity. As a result, the tubing shapes a continuous loop that passes through the intranasal ostium and is unlikely to be dislodged until it has been removed within two to six months. No nasal packing is used unless there is a problem of bleeding or a septoplasty has been done [9].

### 2.4 Histopathological biopsy

All the specimens were sent with the histopathology laboratory in combination with a pathology request. Request form usually contains the clinical and demographic data of the patient [10]. Biopsies derived from the posterior inferior wall of lacrimal sac were processed using fixation in 4% formalin neutral buffer, embedded paraffin, and cutting the specimens at 4–5 μm, then staining mainly with hematoxylin and eosin stain (H&E) and examined under light microscope by the pathologist.

The histopathological features related to chronic inflammation such as inflammatory cell invasion, capillary proliferation and fibrosis were examined then ranked to their intensity utilizing a “chronic inflammation score (CIS)” to assess the degree of chronic inflammation. The features of grading are: 1. The degree of inflammatory cell infiltrates (lymphocytes number, histiocytes, plasma cells) in high power field: mild<50 cells, moderate 50–200 cells, severe>200 cells; 2. The density of fibrosis (the amount of fibrotic tissue in HPF): mild < 25%, moderate 25%–50%, sever >50%; 3. The degree of capillary proliferation (number of capillary vessels) in high power field: mild <5, moderate 5–10, severe>10 [11].

Three characteristics were rated as per chronic inflammation degrees (mild = 1, moderate = 2, and severe = 3) to assess the degree of inflammation of the lacrimal sac. Therefore, the sum of the score for each patient ranged from 3 to 9. Cases were categorized on the basis of this CIS as: chronic inflammation; mild (CIS <3), moderate (3 < CIS < 6) and severe (CIS > 6).

### 2.5 Statistical analysis

The statistical software SPSS v23 was used to perform the analyses (SPSS, Inc, Chicago, Illinois). For all measures, descriptive statistics (means, standard deviations, frequencies, and correlation coefficients) were computed. Determination of P values using the ANOVA test and a χ2 test and Wilcoxon test performed when appropriate. The level of significance was calculated as P < 0.05 was regarded statistically significant, whereas P > 0.05 was regarded statistically insignificant. This study has been performed in line with the STROCSS criteria [12].

## 3 Results

A total of 50 lacrimal sac samples were collected from 50 patients undergoing endoscopic DCR for clinically presumed NLDO acquired in this prospective interventional study. They were 36 women (72%) and 14 men (28%). The average age of patients was 53.4 ± 18.5 with a range between 20 and 70 years (Table 1).

**Table 1:** Age and sex distribution of the studied patients.

Gender		Number	Percent	χ^2^	P
• Males		14	28.0	0.8	0.001*
• Females		36	72.0		
**Total**		50	100		
**Age:**					
• Mean ± SD		53.4 ± 18.5			
• Range		20–70			

χ^2^ = Chi square, *p < 0.001 = highly significant.

The common symptoms of lacrimal duct obstruction were 12 cases (24%) had copious thick mucopurulent discharge coming from the sac, 19 cases (38%) showed mild or moderate mucopurulent discharge, 11 cases (22%) had epiphora with clear tear fluid and 8 (16%) of the patients had swellings over the lacrimal sac with mucoceles-like characteristics (Table 2).

**Table 2:** Symptoms and signs of nasolacrimal duct obstruction.

Symptoms and Signs	Number	Percent
Copious thick mucopurulent discharge	12	24.0
Moderate or mild mucopurulent discharge	19	38.0
Epiphora with clear tear fluid	11	22.0
Swelling over the lacrimal sac (mucocele)	8	16.0
Total	50	100

**Fig. 4:**
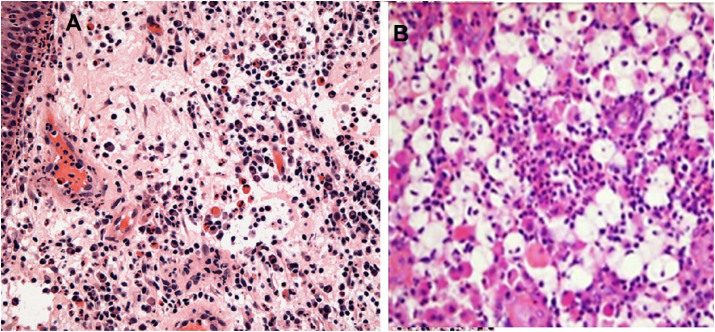
Histological features of; A. a case of chronic inflammation showing lymphoplasmacytic infiltration. B. a case of rhinoscleroma showing macrophages and lymphocytes (H&E stain 100x).

Diagnostic findings of nasolacrimal duct biopsy according to CIS score showed chronic inflammation (Fig. 4) was found in 33 (66%) of patients; 15.2%, 60.6% & 24.2% as mild, moderate & severe CIS grading score, respectively. Chronic dacryocystitis was found in 9 (18%) of patients; 22.2%, 44.4%, & 33.3% as mild, moderate & severe CIS grading score, respectively. Dacryolith with dacryocystitis was found in one patient only. Granuloma (fungal and pyogenic) was found in 4 cases (8%) of patients. Rhinoscleroma was discovered in 2 cases and only one case with neoplasm (squamous papilloma) was observed in severe CIS score (Table 3). There was insignificant relation between these findings (P < 0.05). Intranasal minute adhesions was recorded in 2 cases and another 2 cases of post operative lacrimal sac edema.

**Table 3:** Diagnostic findings of nasolacrimal duct biopsy according to CIS grading.

CIS grade Finding	Mild		Moderate		Severe		Total		P value
	N	%	N	%	N	%	N	%	
Chronic inflammation	5	15.2	20	60.6	8	24.2	33	66	0.09
Chronic dacryocystitis	2	22.2	4	44.4	3	33.3	9	18	0.23
Dacryolith + dacryocystitis	0	0.0	1	100	0	0.0	1	2	0.99
Granuloma	0	0.0	3	75	1	25	4	8	0.06
Rhinoscleroma	0	0.0	1	50	1	50	2	4	0.99
Neoplasm	0	0.0	0	0.0	1	100	1	2	0.99
Total	7	14	29	58	14	28	50	100	

P value was calculated according to Wilcoxon test, p > 0.05 = n.

on-significant.

Histopathologic findings of nasolacrimal duct biopsy according to CIS grading was presented as inflammatory cellular infiltrates that was found in 56%, 30% & 14% in mild, moderate & severe CIS score, respectively. Fibrosis was found in 18%, 20% & 62% in mild, moderate & severe CIS score, respectively. Capillary proliferation was found in 64%, 32% & 4% in mild, moderate & severe CIS score, respectively. Chronic inflammatory signs were found in 64%, 32% & 4% in mild, moderate & severe CIS score, respectively (Table 4). There was insignificant relation between these findings (P < 0.05).

**Table 4:** Histopathologic findings of nasolacrimal duct biopsy according to CIS grading.

CIS grade Finding	Mild		Moderate		Severe		P value
	N	%	N	%	N	%	
Cellular infiltrates	28	56.0	15	30.0	7	14.0	0.08
Fibrosis	9	18.0	10	20.0	31	62.0	0.07
Capillary proliferation	32	64.0	16	32.0	2	4.00	0.06
Chronic inflammatory signs	7	14.0	18	36.0	25	50.0	0.86

P value was calculated according to Wilcoxon test, p > 0.05 = non-significant.

We studied the relation between CIS score and endoscopic DCR outcome and found that patients’ satisfaction was observed in 45 patients (90%), they were 64%, 32% and 4% as mild, moderate and severe CIS score, respectively, while unsatisfied patients were only 5 patients (10%), they were 2 moderate (4%) and 3 severe (6%) as presented in (Table 5). The difference among satisfied and unsatisfied patients was statistically significant (P = 0.002).

**Table 5:** Relation between CIS score and endoscopic DCR outcome.

CIS grade Outcome	Total		Mild		Moderate		Severe		P value
	N	%	N	%	N	%	N	%	
Satisfactory	45	90	32	64	16	32	2	4	0.002*
Unsatisfactory	5	10	0	0	2	4	3	6	

P value was calculated according to Wilcoxon test, *p < 0.01 = significant.

## 4 Discussion

Operation of dacryocystorhinostomy, using an external/endonasal method, was used to surgically treat the obstruction of the nasolacrimal duct. The role of biopsy from the lacrimal system remains contentious from eightieths since neoplasm was discovered in NLD specimens. **Linberg and McCormick** [13] proposed routine nasolacrimal sac incisional biopsy in DCR surgery in 1986. This recommendation was based on a study of 16 biopsies obtained from 15 patients, one of whom (7.5%) had an unexplained primary diagnosis of sarcoidosis due to histology. In a patient with a three-year history of chronic lymphocytic leukemia, another biopsy revealed lymphoma. From that date, incisional nasolacrimal sac biopsy was recommended as a routine in DCR surgery by many surgeons, irrespective of appearance or risk factors [[13], [14], [15], [16]]. The occurrence of lacrimal system neoplasms in various age groups from 20 to 90 years was reported, and is invasive to adjacent tissues and has a high recurrence rate; it is commonly misdiagnosed as an obstruction or inflammation then accidently detected during pathology examination which is the most accurate investigation for challenging neoplastic lesions [[16], [17], [18], [19], [20]].

The high resolution of nasal endoscopes enables for outstanding visualization of the interior sac through endoscopic DCR that can provide clinicians with valuable knowledge on lacrimal pathology that has gone undetected [21].

In such instances, the significance of biopsy of the lacrimal system and histopathological examination of the sac wall during DCR surgery is undeniable [22]. So, the present study was performed manly to determine the significance of the NLD biopsy and histopathologic examination during endoscopic DCR. It included 50 lacrimal sac specimens from patients underwent endoscopic DCR for NLDO treatment. They were 36 females (72%) and 14 males (28%). The mean age of patients was 53.4 ± 18.5 with a range between 20 and 70 years.

In **Banks et al.** [21], their neoplastic cohort had an average age of >70 years, while granulomatous disease patients had an average age of <45 years. The granulomatous cohort's age range was 31–63 years, which corresponded to the well-known bimodal sarcoidosis distribution [23,24]. In patients <50 years of age, the idiopathic NLDO is less frequent, and secondary reasons in these patients should be considered [25]. However, **So et al.** [16] did not find significant difference in prevalence of tumor according to race and sex.

The common symptoms of lacrimal duct obstruction were 12 cases (24%) had a lot of thick mucopurulent discharge coming from the sac, 19 cases (38%) showed mild or moderate mucopurulent discharge, 11 cases (22%) had epiphora with clear tear fluid and 8 (16%) of the patients had swellings over the lacrimal sac with mucoceles-like characteristics.

Diagnostic findings of nasolacrimal duct biopsy according to CIS score showed chronic inflammation was found in two thirds of patients; Chronic dacryocystitis was found in 18% of patients and dacryolith with dacryocystitis was found in one moderate patient. Granuloma was found in four patients (8%). Rhinoscleroma was detected in 2 cases (4%). Only one case had neoplasm (2%) in our study which coincides with the previous literatures that reported 0%–2.3% [ [2,6,7,[26], [27], [28], [29]]]. However, the unexpected rate of neoplasms and granulomatous pathology in **Banks et al.** [21] series was only 0.46% (n = 3 of 728 lacrimal sac specimens). They added that the patient's history, endoscopic assessment, CT imaging and intraoperative results were not indicative of neoplastic or granulomatous inflammation in all three unexpected instances. Neoplasms were present in 0.9% of all samples in their study that is slightly lower than the recorded occurrence of lacrimal neoplasms (1.43%) [23,24]. In this cohort, the prevalence of granulomatous pathology (1.0%) was slightly lower than the estimated incidence of lacrimal sac granulomas (2–8%) [30].

In this study, in 8% of the patients, granuloma was discovered which was similar to many studies as described above. In contrary to these results, **Amin et al.** [2] reported in their series of 294 NLDO patients, non-granulomatous findings by histopathological specimens. In their study, none of the patients had normal histology, and all 33 patients had non-specific lacrimal pathology, which included different degrees of chronic inflammation, but no specific lacrimal system pathology was found.

Of the 316 patients, 377 DCR lacrimal sac biopsies were reviewed by **Anderson et al.** [8]. There were 17 neoplasms (4.5%) in total, of which eight (2.1%) were undetected preoperative. Eight neoplastic samples (1.34%) were discovered in a prospective sequence of 599 patients who had external DCR in 2012, six of whom had no clinical symptoms, signs, or intraoperative appearance indicative of a potential underlying lacrimal sac tumor [26]. **Rauter et al.** [29] conducted a retrospective study of 218 DCR patients in 2018 and discovered five neoplasms (2.3%), all of which have been previously undiagnosed. Although the occurrence of neoplasm in such series is relatively low, these results do tend to support the use of lacrimal system biopsy during DCR.

In a large coherent study by **So et al.** [16] on 1266 people (1619 eyes), four were diagnosed with malignant tumors diagnosed as lymphoma, two of them had a palpable mass in the medial canthum before surgery, while that other two had no additional sings than NLDO or in the CT imaging, they came to the hospital for treatment of epiphora.

Histopathological examination can specify the nature and severity of different inflammatory lesions [31,32], our findings of nasolacrimal duct biopsy in this study according to CIS grading was presented as inflammatory cellular infiltrates that was found in 56%, 30% & 14% in mild, moderate & severe CIS score, respectively. Fibrosis was found in 18%, 20% & 62% in mild, moderate & severe CIS score, respectively. Capillary proliferation was found in 64%, 32% & 4% in mild, moderate & severe CIS score, respectively. Chronic inflammatory signs were found in 64%, 32% & 4% in mild, moderate & severe CIS score, respectively. There was insignificant relation between these findings (P < 0.05).

In agreement of our study, So et al. [16] found in cases most of the biopsy results were chronic inflammation and fibrosis. However, the preoperative and intraoperative malignancies were unpredictable unless diagnosed by biopsy or imaging study. Although incidence of malignancy is low, poor prognosis may occur in neglected cases or late biopsy, while early detection by biopsy had been cured when treated early. The same was achieved by Banks et al. [21] in their endoscopic DCR series as they found most of lacrimal biopsies were either inflamed or normal-appearing sac mucosa, which was compatible with other DCR surgery results [8,26,28,33,34].

**Amin et al.** [2] applied the CIS framework to the previously mentioned findings, 27 (81.8%) of the instances with chronic inflammation displayed moderate CIS ranged between 4 & 6, where 4 (12.1%) of cases had extreme inflammatory shifts with CIS of 7. Mild degree was observed in 2 cases (6.06%) with CIS of 3, which was similar to our results.

On the other hand prior to DCR surgery, **Golan et al**. [35] studied the clinical history and CT records of 47 patients with a diagnosis of NLDO. Preoperative CT scanning revealed 4 patients (8%) with unpredicted pathologies, and imaging was recommended in select cases to aid in the diagnosis of lacrimal system lesions that were previously unsuspected.

Preoperative CT imaging can reveal areas of persistent sinusitis overlying the lacrimal sac, allowing for the detection of previously undetected neoplasms that can help determine not only the cause of the patient's nasolacrimal duct obstruction, but also the best post-surgery medical treatment plan. Finally, a formal cost-utility analysis will be needed before recommending universal preoperative CT imaging prior to DCR [20]. In their research, intraoperative endoscopic results able to alert the surgeon to apparent pathology in CT scan in 2 out of 7 neoplastic instances, and 3 of them have had abnormal pathology via histopathology. The results varied from a subtle thickening or edematous shape of the lacrimal system to a discrete mass inside the sac. The other four patients proved to be malignant by histopathological biopsy.

The contiguous anatomical relation and shared histological characteristics of the nasal lacrimal system and the nasal mucosa have been cited in different publications to support the association among lacrimal sac and sinonasal sarcoidosis [23,24]. Particularly, adjacent sinonasal sarcoidosis was found in 62.5% of neoplastic patients in the **Banks et al.** [21] cohort. The fact that two out of every eight patients had a “cobblestone” appearance of the nasal mucosa or an exposed lacrimal sac endorsed the value of intraoperative evaluation. While given the systemic nature of the disease, one could expect a higher prevalence of bilateral disease in sarcoid patients, the small number of sarcoid patients in this research prevented a conclusive statistical conclusion.

We studied the relation between CIS score and endoscopic DCR outcome and found that patients’ satisfaction was observed in 45 patients (90%), they were 64%, 32% & 4% as mild, moderate & severe CIS score, respectively, while unsatisfied patients were only 5 patients (10%), they were 2 moderate (4%) and 3 severe (6%) as presented in table (5). There was a statistically significant difference between satisfied and unsatisfied patients (P = 0.002).

Similar to our study, **Amin et al.** [2] statistically analyzed the histopathological changes and severity of chronic inflammation to patients with satisfactory and unsatisfactory outcome, and found statistically significant values between the two groups.

The cost of biopsy is not high. It doesn't take much time for high surgery and it does not require special skills. It has no effect on the success rate of surgery and the risk of biopsy itself is not great. Therefore, in all patients, the intraoperative biopsy is not to be missed [16]. Attention should be taken place in patients with abnormal findings and weeping requires more attention [33].

From the above, we supported biopsy during endoscopic DCR. Other studies support selective biopsy of the lacrimal system during DCR [2,6,7,14,21,33]. In a group of 193 successive lacrimal biopsies through endoscopic DCR, **Merkonidis et al.** [14] identified a single case of neoplasm (transitional cell papilloma), subsequently, a literature review was conducted and it was noticed that 7 (0.5%) of patients in a composite series of 7 trials of 1294 samples had no pre-or intra-operative suspicion of pathology. It was discovered that one of such instances was malignant (0.08%). Lacrimal sac wall biopsy, according to the researchers, is a low-yield technique that can only be used if a disease other than simply chronic inflammation is suspected. **Bernardini et al.** [33] stratified 302 lacrimal sac biopsies depending on the risk factors and came to the conclusion that lacrimal sac biopsy should only be done in DCR patients with a positive history of systemic disease or an abnormally presenting lacrimal sac during surgery.

The main limitation in this study is the very low number of patients as this kind of researches requires very large coherent studies to detect the pathology for more accurate statistics. Another limitation is the lack of control group for comparison of the results. The research lacked the necessary power to conduct multivariate studies to look at the effects of age and other potentially influential variables like sex and comorbidities on these results. Ultimately, the follow-up period was not mentioned in this study due to variable time of follow-up.

## 5 Conclusion

From this study and previous reports, we concluded that it is highly advisable to perform tissue biopsy and histopathological examination in all cases of endonasal DCR for detection of unexpected underlying pathology and early treatment for better prognosis. Also we suppose that the biopsy from lacrimal sac is mandatory in recurrent cases or unexplained pathology.

## Funding sources

This study did not receive any funding from public or private sectors.

## Ethical approval

Ethical approval was obtained from the ethical committee of Al-Azhar University Hospitals.

## Informed consent

Electronic and written informed consent was obtained for publication of this study.

## Provenance and peer review

Not commissioned, externally peer-reviewed.

## Author contribution

Study concept or design: IE, WFI, AAI, MEA, AH, MHA,

Data collection: IE, MHA, WFI, ARM, MEA

Data interpretation: IE, WFI, AH, AAI, MEA, ARM

Literature review: IE, WFI, MHA, MEA, AH, AAI.

Data analysis: IE, WFI, AH, MEA, ARM.

Drafting of the paper: ALL.

Editing of the paper: ALL.

Manuscript revision: ALL

## Registration of Research Studies

ClinicalTrials.gov Identifier: **NCT04793230**

## Guarantor

Dr. **Ibrahim Eldsoky**

## Declaration of competing interest

The authors declare no competing interests.
